# In-vivo Kinematics of the Cervical Spine in Frontal Sled Tests

**DOI:** 10.5539/gjhs.v5n3p115

**Published:** 2013-02-18

**Authors:** Christoph Dehner, Sylvia Schick, Wolfram Hell, Peter Richter, Michael Kraus, Michael Kramer

**Affiliations:** 1 Department for Trauma, Hand, Plastic and Reconstructive Surgery, University of Ulm, Ulm, Germany; 2 Institute for Legal Medicine, Ludwig Maximilians University of München, München, Germany

**Keywords:** frontal impact, cervical spine kinematics, volunteer sled test, risk of injury

## Abstract

The description of cervical spine motion and the risk to sustain a cervical spine injury in traffic accidents is mainly based on rear-end collisions. The knowledge about frontal collisions is comparable low. Therefore the objective of this exploratory study was, to describe the in-vivo cervical spine motion and acceleration during simulated frontal sled collisions and to identify sequences of motion in which the risk of injury is increased.

A frontal collision with a speed change of 10.2km/h was simulated in a sled test with ten volunteers. Cervical spine kinematics was assessed by the simultaneous analysis of the angular head motion and acceleration as well as the simultaneous analysis of the relative motion and acceleration between the head and the first thoracic vertebral body.

The motion sequence was divided into five phases. The combination of peak values of the angular head acceleration to ventral and the relative horizontal head acceleration to dorsal between the time period of 90ms and 110ms (early flexion phase) included – potential injury generating – shear forces. Although a hyperflexion (late rebound phase) as injury pattern didn’t occur, dorsal soft tissue injuries due to eccentric muscle-sprain could not be ruled out completely.

In conclusion the study showed under simulated test conditions that during the early flexion phase and the late rebound phase, acceleration and movement pattern occur that could lead to cervical spine injuries.

## 1. Introduction

Since Gay and Abbott (1953) first described the whiplash phenomenon, the mechanism, which causes the cervical spine injuries subsequent to car-to-car collisions has been intensively explored. Throughout the last twenty years, cervical spine motion and intersegmental applied forces have been intensively analyzed in post mortem human spine specimens and volunteer tests, focusing on rear-end collisions (Cholewicki et al., 1998; Luan et al., 2000; Panjabi et al., 1998). These studies have identified an initial transient S-shape of the cervical spine in response to a rear-end impact, in which the more cranial motion segments undergo flexion, coupled with hyperextension in the more caudal segments.

By comparison, the mechanisms of injury operating during frontal collisions remain largely unstudied (Kumar, Narayan, & Amell, 2003). The first systematic evidence of damage to structures in the cervical spine after frontal collisions was reported by Bucholz, Burkhead, and Graham (1979) from post-mortem examinations. Subjects sustaining high-impact trauma exhibited ruptures of the dorsal ligaments. Further studies of post-mortem human subjects (PMHS) sustaining frontal collisions have confirmed injury to dorsal ligament structures (Crowell, Shea, & Edwards, 1993).

Panjabi et al. (2004) were able to further characterize these injuries in PMHS sustaining frontal collisions. After the simulated frontal collision they showed in an anatomical investigation that the greatest elongations of both the supra- and interspinous ligaments and of the ligamentum flavum occurred at the cervical level C3/4 as hint for the main location of the injury. These biomechanical studies were based on a hyperflexion motion of the cervical spine occurring during frontal collisions.

The first *in-vivo* frontal collision tests were conducted between 1970 and 1990 and predominantly involved military personnel in studies of strain in aircraft carrier pilots (Ewing, Thomas, Lustick, & Becker, 1975; Grunsten, Gilbert, & Mawn, 1989). The simulated changes in velocity (ΔV) of the vehicle were relatively high with an average ΔV of 40-50 km/h. As low-velocity simulations are defined with a threshold lower than 15 km/h, these tests could not be considered as low-velocity simulations in the strict sense.

Subsequent *in-vivo* studies of frontal collision events have similarly failed to provide an exact characterization of the biomechanical mechanisms involved in these occurrences (Kullgren, Krafft, Nygren, & Tingvall, 2000; Larder, Twiss, & Mackay, 1995). In an analysis of real frontal crash events in Sweden documented by an on-board black box, Kullgren et al. (2000) reported that with an average ΔV of 22 km/h frontal collisions were associated with injury thresholds that were twice as high compared with rear impacts. The authors explained this difference biomechanically by the simultaneous forward motion of the torso and head over a significantly longer distance before reaching the seatbelt. Further biomechanical studies to more precisely characterize the kinematics of the cervical spine during frontal collisions were not identified in the literature.

This paucity of data is astonishing given the fact that frontal collisions are cited in the literature as the most frequent type of motor vehicle accident, making up about 60% of these occurrences (O’ Neil, Lund, Preuss, & Zuby, 1994). In addition, these accidents are the cause about one-third of all cervical spine injuries (Kullgren et al., 2000). Thus, the exact kinematic behaviour of the cervical spinal column during crash situations has been adequately described only for rear collisions. This explains why it is still unknown which time period during the crash situation in frontal collision is associated with the highest risk of cervical spine injury. Therefore the objective of this exploratory study was, to describe the in-vivo cervical spine motion and acceleration during simulated frontal sled collisions and to identify sequences of motion in which the risk of injury is increased.

## 2. Methods

### 2.1 Subjects

The work has been approved by the local ethics committee. The people were invited to participate in the study by an invitation letter, which was distributed in the University of Ulm. Thereby the intended experiment was outlined. In case of interest to participate in the study a contact phone number was given. N=13 people responded to the invitation to participate. The participants were asked about their medical history. Additionally an orthopaedic surgeon performed a clinical examination. The clinical examination consists of the investigation of muskuloskeletal and osseous spinal triggerpoints, the neurological examination of the peripheral nervous system and the determination of the range of motion of the spine. Exclusion criteria were a history of whiplash injury of the cervical spine, neurological or psychiatric disease, functional impairments of the cervical spine or cervical spine pain. n=10 participants could be selected after proofing the exclusion criteria. The participants were informed about the exact test procedure and gave written consent to participate in the study.

### 2.2 Experimental Design

**Figure 1:**
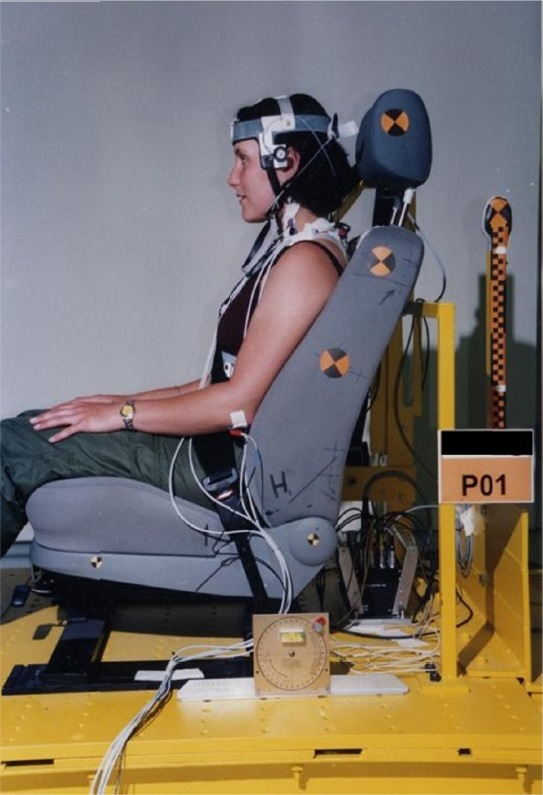
Side view of test sled

For the frontal collision simulation we used a standard automobile seat (VW Passat, 2000 model, VW corporation, Wolfsburg, Germany) anchored to a sled platform (Figure 1). The seat sled was accelerated over a length of 20 meters towards the fixed iron barrier. With each participant one sled test was performed.

Measurement of the sled acceleration was performed using a sensor (Endevco 2262, +/- 200g, uniaxial x-direction, CFC 60, Endevco Corporation, San Juan Capistrano, USA). The sled acceleration is characterized by a triangular impulse. The calculation of Δv was performed on the basis of the CFC180 filtered sled acceleration. The mean acceleration of the seat sled was 2.68 g and the mean Δv was 10.2 km/h (Table 1).

**Table 1:** Characteristics of the sled test acceleration (N=10)

	Mean sled acceleration [g]	Maximal sled acceleration [g]	Duration [ms]	Δv [km/h]
Minimum	2.45	3.57	100	9.9
Maximum	3.27	4.89	110	12.7
Median	2.68	4.00	110	10.2
Mean	2.76	4.05	106	10.4
Standard deviation	0.21	0.35	5	0.8

Δv: change of velocity

After positioning the subjects on the test sled, head restraints were adjusted to the best possible vertical position, which means that the upper edge of the head-restraint was aligned with the vertex of the head of each subject (Figure 1). A horizontal adjustment was not possible. The initial horizontal distance from the head to the head restraint ranged from 40 to 90mm (median 60mm). In addition, all subjects were secured with a three-point seat belt in passenger position. The backrest was fixed and checked after each test for remaining in 25° backrest angle to seat cushion.

### 2.3 Measurement Technique, Data Recording and Processing

The data mentioned below were recorded for all subjects from –800ms to +800ms, with “0” defining the time of the trigger response. Data recording and processing according to Society of Automotive Engineers ((SAE) J211/1; 1995) was performed with Diadem® 8.0 (National Instruments Germany GmbH, Munich, Germany).

#### 2.3.1 Motion Data and Visual Documentation

The experiments were recorded with a stationary LOCAM high speed camera (Visual Instrumentation Corporation, USA) and subsequent digitized with 100 images/s. The motion data were documented based on markings of the center of gravity of the head (surface projection ca. 1.5 cm ventrally and cranially to the most cranial point of the external acoustic meatus), the Frankfort plane (defined as the inferior margin of the osseous orbit and the upper margin of the external ear canal) and the first thoracic vertebra. Tracking of the target in the digitized film was performed in 10ms steps for the first 300ms. The motion was calculated on the basis of the sled-related coordinate system (x-axis (positive forwards), the y-axis (positive to the left) and the positive z-axis extends perpendicularly upwards). The data were smoothed prior to further processing, using a third order spline which is at least square error optimized (Woltring, 1986).

#### 2.3.2 Accelerations

To measure the angular head acceleration also the rotation rate sensor (Endevco 7302, 5000rad/sec², piezoresistive (Endevco Corporation, San Juan Capistrano, USA)) was fixed in a position close to the projected centre of gravity of the head.

In order to measure the T1 acceleration (acceleration of the first thoracic vertebral body), a two-axial linear accelerometer (Endevco 7264, +/- 200g (Endevco Corporation, San Juan Capistrano, USA)) was mounted on a pliable metal plate, which was padded with tape, adjusted to the contour of the subject’s back and attached directly to the skin above the spinal process of the first thoracic vertebra. The starting position of the T1 sensor above the spinal process of the first thoracic vertebral body defined the sensor-related coordinate system. The positive x-axis pointed in ventral direction, perpendicular to the body surface, and the positive z-axis, which was perpendicular to the x-axis pointed in cranial direction. The accelerations are not compensated for gravity, as they are automatically set to zero prior to the test.

The measurement of the horizontal head acceleration was performed using a three-axial linear accelerometer MSC 123 sensor (+/- 100g (Micro-epsilon Messtechnik GmbH & Co. KG, Ortenburg, Germany)). The accelerometer was attached to the subject via a head harness with which the sensor could be positioned as close as possible to the projected centre of gravity of the head. Analogue to the motion data the anatomical head coordinate system was based on the Frankfort-plane. The relative acceleration between the head and T1 in x-direction was calculated from these accelerations.

### 2.4 Analysis

Due to the high costs for the performance of each experimental sled test and the given financial budget for the study procedure, the maximal sample size of ten participants was already fixed at the beginning of the study. Therefore no power analysis was performed. The analysis of the motion and acceleration curves was performed descriptively for each participant. Generally, the start of acceleration or motion was defined as the time at which 10% of the subsequent maximum/minimum was reached or the zero-crossing when a change of sign occurred. The following biomechanically relevant parameters of the motion and acceleration curves were ascertained chronologically concerning the amplitudes and the time of occurrence (Table 2). For final data presentation the statistical values – minimum, maximum, median, mean and standard deviation – was calculated out of all 10 samples. The definition of the motion phases was performed on the basis of the time of occurrence of the calculated motion parameters.

**Table 2:** Definition of biomechanically relevant kinematic events in the motion and acceleration curves

Parameter	Definition
Angular head movement	
Beginning of head flexion	10% of the maximum
Maximal head flexion	Maximum
Relative horizontal head movement	
Beginning of ventral head translation	10% of the maximum
Maximal ventral head translation	Maximum
Angular head acceleration	
Beginning of ventral angular head acceleration	10% of the maximum
Maximal ventral angular head acceleration	Maximal positive peak
Beginning of dorsal angular head acceleration	Zero crossing
Maximal dorsal angular head acceleration	Maximal negative peak
Relative horizontal head acceleration	
Beginning of dorsal horizontal head acceleration	10% of the maximum
Maximal dorsal horizontal head acceleration	Maximal negative peak
Minimal dorsal horizontal head acceleration	Relative minimal negative peak

## 3. Results

### 3.1 Subjects

Ten subjects (seven men, three women) aged 20 to 47 years (median: 35 years) without prior structural injuries to the spine participated in the study. Subjects’ height ranged from 170 to 191 cm (median: 180 cm), their mass was 61-110 kg (median: 83.5 kg), their neck circumference ranged from 26 to 49 cm (median: 40 cm) and their thorax circumference ranged from 80 to 112 cm (median: 102 cm).

### 3.2 Description of the Motion Phases

**Figure 2:**
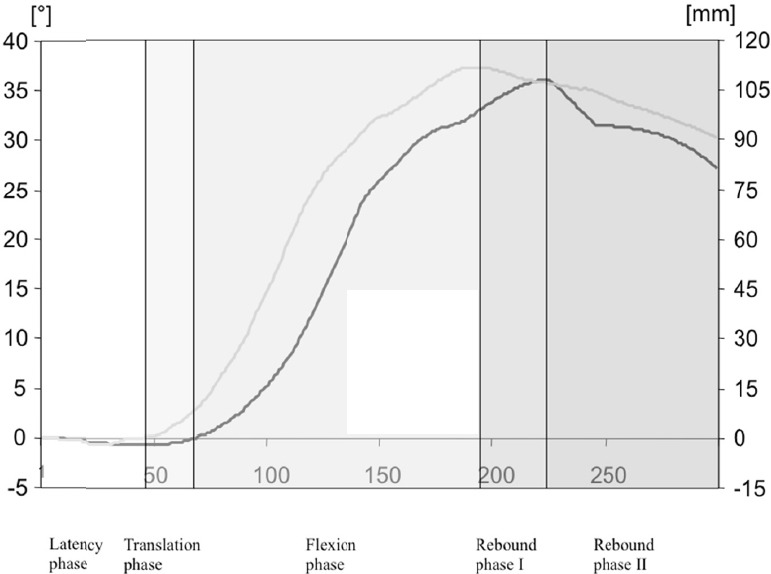
Median curves of the motion parameters and definition of the motion phases on the basis of the most relevant motion parameters Black curve: relative horizontal head movement [mm]; grey curve: angular head movement [°]

The motion sequence was divided into five phases (Table 3 and Figure 2). This was reproducible for all subjects. In all subjects, the maximal motion amplitudes during the frontal collision fell within the physiological limits determined at the beginning of the study (Table 4). In the latency phase (0ms-44ms) the head initially remained in its baseline position. In the translation phase (44ms-68ms) the ventral translation of the head began without a rotational component after a median of 44ms (25ms-64ms). In the flexion phase (68ms-196ms) additionally to the isolated translation ventral flexion of the head starts after a median of 68ms (60ms-80ms). A head to headrest contact did not take place at any time. After a median of 196ms (175-233ms) the maximal ventral head translation (median: 112mm, 62-132mm) was reached (Table 4). During the rebound phase I (196ms-223ms) the head reached its maximal head flexion (median: 36.2°, 21.2-48.2°). In the rebound phase II (223ms-300ms) the maximal ventral translational motion of the head and the head flexion were both decreasing slightly. These results were reproducible for all subjects.

**Table 3:** Definition of the motion phases on the basis of the most relevant motion parameters

Motion phase	Parameter
Latency phase	
Beginning	Beginning of the sled acceleration
End	Beginning of the ventral head translation
Translation phase	
Beginning	Beginning of the ventral head translation
End	Beginning of the head flexion
Flexion phase	
Beginning	Beginning of the head flexion
End	Time of maximal ventral head translation
Rebound phase I	
Beginning	Time of maximal ventral head translation
End	Time of maximal head flexion
Rebound phase II	
Beginning	Time of maximal head flexion
End	End of data presentation (300ms)

**Table 4:** Amplitudes of the Motion and Acceleration Parameters as defined in Table 2

Parameter	Minimum	Maximum	Median	Mean	Standard deviation	
Angular head movement [°]					
Maximal head flexion	21.2	48.2	36.2	35.0	7.1
Relative horizontal head movement [mm]					
Maximal ventral head translation	62	132	112	110	20
Angular head acceleration [rad/s²]					
Maximal ventral angular head acceleration	110	367	177	207	69
Maximal dorsal angular head acceleration	231	425	260	308	82
Relative horizontal head acceleration [g]					
Maximal dorsal horizontal head acceleration	4.1	6.2	5.0	5.1	0.6
Minimal dorsal horizontal head acceleration	0.4	1.7	1.2	1.1	0.4

### 3.3 Analysis of Kinematic Phases in Relation to Motion and Acceleration Parameters

**Figure 3:**
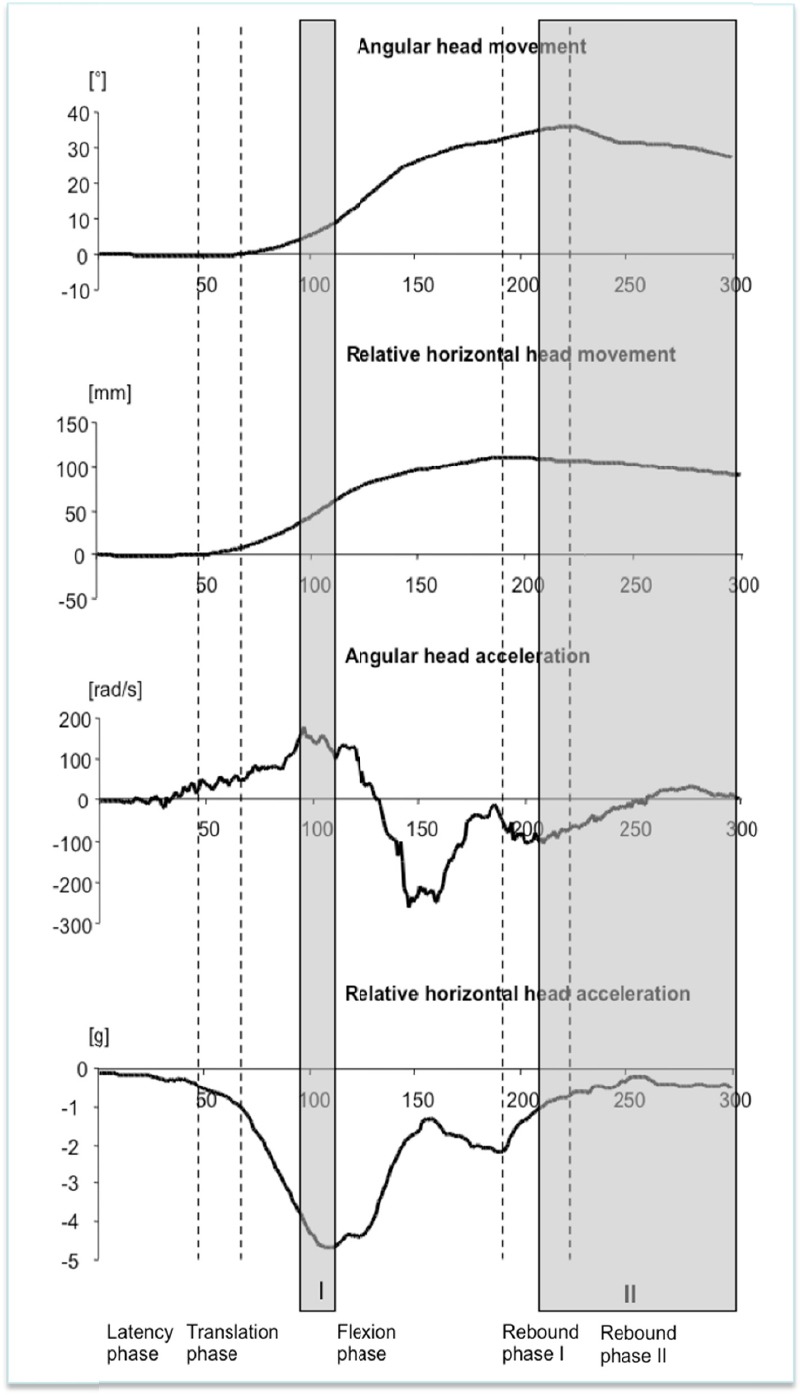
Median curves of the motion and acceleration parameters Grey box I: Time period between 90ms and 110ms with maximal contrary acting forces; Grey box II: Parallel deceleration of head flexion and ventral head translation

Analysis of all four parameters (angular head movement, angular head acceleration, relative horizontal head movement and relative horizontal head acceleration) permits a more exact impression of force impact and time of its occurrence (Tables 4 and 5, Figure 3). The following results were reproducible for all subjects.

**Table 5:** Time of occurrence of the motion and acceleration parameters in [ms] as defined in Table 2

Parameter	Minimum	Maximum	Median	Mean	Standard deviation
Angular head movement					
Beginning of head flexion	60	80	68	69	6
Time of maximal head flexion	192	239	223	212	19
Relative horizontal head movement					
Beginning of ventral head translation	25	64	44	43	12
Time of maximal ventral head translation	175	233	196	203	21
Angular head acceleration					
Beginning of ventral angular head acceleration	21	46	31	32	8
Time of maximal ventral angular head acceleration	73	138	96	102	21
Beginning of dorsal angular head acceleration	120	144	131	131	7
Time of maximal dorsal angular head acceleration	144	164	149	152	6
Relative horizontal head acceleration					
Beginning of dorsal horizontal head acceleration	26	75	49	53	16
Time of maximal dorsal horizontal head acceleration	97	134	106	108	10
Time of minimal dorsal horizontal head acceleration	143	168	156	158	7

In the latency phase (0ms-44ms), initially isolated ventral angular head acceleration occurred after a median of 31ms (21-46ms). In the translation phase (44ms-68ms) the relative horizontal head acceleration started in dorsal direction after a median of 49ms (26-75ms). There is only a slight relative ventral translation movement and, consequently, the cervical spine is almost in its initial position.

In the flexion phase (68ms-196ms) the maximal braking effect of the seat belt occurred after a median of 90ms (81ms-99ms). As the horizontal head movement had been slowed down over the whole flexion phase (maximal deceleration – median: 106ms; 97ms-134ms; minimal deceleration – median: 156ms; 143ms-168ms), the head flexion was firstly accelerated (median: 96ms; 73ms-138ms) until a deceleration process after a median of 131ms (120ms-144ms) was initiated. Therefore in the time period between 90ms and 110ms maximal contrary acting forces could be found (grey box I, Figure 3). In this time period the ventral angular head acceleration (median: 177rad/s², 110-377rad/s²) and the dorsal horizontal head acceleration (median: 5.0g, 4.1-6.2g) reached their peak values. This was followed by the events of maximal dorsal angular head acceleration (median: 260rad/s², 231-425rad/s²) after a median of 149ms (144ms-164ms) and the minimum of dorsal horizontal head acceleration (median: 1.2g, 0.4-1.7g) after a median of 156ms (143ms-168ms).

In the rebound phase I and II (196ms-300ms), the parallel deceleration of head flexion and ventral head translation occurred. In this phase, there were constant but low acceleration values over a long period still acting (grey box II, Figure 3). After the maximal ventral translation (median: 112mm, 62-132mm) and the maximal flexion amplitude (median: 36.2°, 21.2-48.2°) were reached, a slight backwards movement occurred.

## 4. Discussion

Yet, for the improvement of car occupant safety systems, it is important to produce data that are related as closely as possible to the in-vivo situation (Kaneoka, Ono, Inami, & Hayashi, 1999). As the data resulting from cadaver models are limited in their significance for the in-vivo situation, volunteer tests are necessary. While volunteer tests capture actual cervical spine kinematics, they are limited by the fact that the forces acting on the cervical spine can only be determined approximately. Direct force measurements are not feasible and this requires reference to indirect measurement parameters in the form of acceleration data (Matsushita et al., 1994).

Up to now, inadequate use has been made of sled tests with human subjects in the low velocity range for the purpose of investigating the kinematic processes and injury risk associated with frontal collision. As a result, the ability to compare the results of the present study with previous reports in the literature is limited. The present study described the frontal impact event at a ΔV of 10.2 km/h on the basis of the recorded motion and acceleration data in volunteers. The parallel analysis of the angular head acceleration and the relative horizontal acceleration of the head shows that the two components of acceleration do not operate simultaneously.

The latency phase begun with early ventral angular head acceleration without a relative horizontal head acceleration. After that a horizontal acceleration of T1 in ventral direction with a simultaneous relative dorsal acceleration of the head rose up while the ventral angular acceleration of the head was ongoing. While the study of Kumar et al. (2003) provided no motion data, the onset of the resulting head acceleration at a sled acceleration of 1.4g was reported at 35.6 ms. These data correspond well with the findings of the present study, with measured onset of ventral head acceleration at 31 ms and of dorsal horizontal acceleration of the head at 49 ms. The result of this was, first, a relative ventral translation of the head without rotational component, which is secondly accomplished by a flexion movement of the head. Up to this point the reacting forces were comparable low, so that no relevant injury risk could be stated

Distinct from rear collisions, dorsal translation or extension movements did not occur at any point in time and could thus be excluded as causes of injury. There was also no contact between the head and the headrest, which confirmed the assessment of Walz (1994) that the headrest plays no role in frontal collisions.

After that the described acceleration pattern was ongoing, reaching peak values between the time period of 90ms and 110ms. As the amplitude of the relative horizontal head acceleration corresponded to the peak values during a rear-end collision, the peak values of the angular acceleration rose up to threefold compared to the rear-end collision (Dehner et al., 2007). The comparable peak values of the head acceleration were in the frontal sled test study of Deng, Begeman, Yang, Tashman, and King (2000) with 6g to15g even higher. This fact and the above mentioned narrow time-corridor illustrates that the reacting forces reached in this time period an unfavourable constellation. Thereby the angular head acceleration could be interpreted as distraction force of the dorsal structures and compression force of the ventral structures of the cervical spine, whereas the relative horizontal acceleration of the head represented shear forces in dorsal direction at the same time. The combination of compression forces in the ventral structures of the cervical spine and dorsal directed shear forces had been assumed to be associated with a high potential for causing injury to the discs (Ito et al., 2005). However, as the head has not yet reached a significant deflection, injury mechanisms such as stretching of dorsal ligaments or capsules are unlikely. Further in-vivo data, especially with regard to the chronological occurrence of peak accelerations, are not available for comparison.

In this study the maximal braking effect of the seat belt occurred after a median of 90ms (81ms-99ms), close before the above described risky time corridor. Kullgren et al. (2000) considered the deceleration of the body in its forward motion by the onset of the seat belt effect to be the main risk factor. In addition Siegmund, Chimich, Heinrichs, DeMarco, and Brault (2005) demonstrated that maximum motion and acceleration vary significantly in relation to the attachment and tension of the seat belt. For example, with lax seat belt tension, there was an increase in both ventral motion and ventral acceleration amplitudes of the thorax. By contrast, Walz (1994) postulated that, in the absence of head impact and limited vehicular damage, cervical spine injury would occur only in subjects with pre-existing damage to the cervical spine or significant head rotation during the crash event.

In the rebound phase II the risk of injury must be assessed together with the increasing ventral motion of the head. Unlike during the previous movement, external force applications resulting in a deceleration of the head were mainly absent. Therefore deceleration forces have to be extinguished by active muscle tension or increasing of the soft tissue resistance like the stretched dorsal ligamentous and capsular structures. Panjabi (1992) found out that at the end of the physiological range of motion – in the elastic zone of ligaments – only a small force increase is necessary to cause plastic distortion. Larder et al. (1995) therefore assumed that in frontal collision without a head contact the flexion movement of the head solely causes the injury. The maximum median flexion of the head observed in the present study was 28.9°, which remains within the normal physiological range of motion. Keeping in mind the assumptions of McPherson, Schorck, and Faulkner (1996) and Kumar, Ferrari, and Narayan (2005), however, the combination of such injury with an excessive increase in muscle activity cannot be ruled out as a cause of patients’ complaints. This relationship should be addressed in further studies focusing on muscular reaction time and its interaction with cervical spine kinematics.

The results of the sled tests performed in this study are simulating a passenger position, which could be different of the driver position in the case of a direct impact due to the steering wheel. If no direct impact with the steering wheel occurs, minor changes in the kinematic behaviour could not be ruled out due to the different position of the arms and a possible stabilizing effect of the torso. Up to now the investigation of differences of the kinematic behaviour between the driver and passenger position in frontal collisions has not performed. As further limitation of the study the existence of a small sample size has to be mentioned. This is caused by the quite complex and expensive experimental setup, which didn’t allow larger test series. As the maximal sample size of ten participants was already fixed at the beginning of the study, no power analysis was performed. Nevertheless this fact leads to a reduction of the validity of the study results and a lack of power for generalizing the conclusions. Therefore the data of this study are descriptive and have to be confirmed in further studies. Until then the data of this study have to be assessed as preliminary results.

## 5. Conclusions

The study shows that during the early flexion phase and the late rebound phase kinematic constellations exist that could lead to cervical spine injuries. In the flexion phase contrary acceleration applications could lead to ventral shear injuries of the disco-ligamentous structures. As important study result the maximal ventral head movement did not exceed the physiologic range of motion excluding a hyperflexion movement as key injury pattern. Nevertheless the occurrence of dorsal soft tissue injuries due to eccentric muscle-sprain in the late rebound could not be ruled out completely.
